# How Prevalent Is Object-Based Attention?

**DOI:** 10.1371/journal.pone.0030693

**Published:** 2012-02-13

**Authors:** Karin S. Pilz, Alexa B. Roggeveen, Sarah E. Creighton, Patrick J. Bennett, Allison B. Sekuler

**Affiliations:** 1 Department of Psychology, Neuroscience and Behaviour, McMaster University, Hamilton, Ontario, Canada; 2 Sheridan Elder Research Centre, Sheridan Institute of Technology and Advanced Learning, Oakville, Ontario, Canada; CNRS - Université Claude Bernard Lyon 1, France

## Abstract

Previous research suggests that visual attention can be allocated to locations in space (space-based attention) and to objects (object-based attention). The cueing effects associated with space-based attention tend to be large and are found consistently across experiments. Object-based attention effects, however, are small and found less consistently across experiments. In three experiments we address the possibility that variability in object-based attention effects across studies reflects low incidence of such effects at the level of individual subjects. Experiment 1 measured space-based and object-based cueing effects for horizontal and vertical rectangles in 60 subjects comparing commonly used target detection and discrimination tasks. In Experiment 2 we ran another 120 subjects in a target discrimination task in which rectangle orientation varied between subjects. Using parametric statistical methods, we found object-based effects only for horizontal rectangles. Bootstrapping methods were used to measure effects in individual subjects. Significant space-based cueing effects were found in nearly all subjects in both experiments, across tasks and rectangle orientations. However, only a small number of subjects exhibited significant object-based cueing effects. Experiment 3 measured only object-based attention effects using another common paradigm and again, using bootstrapping, we found only a small number of subjects that exhibited significant object-based cueing effects. Our results show that object-based effects are more prevalent for horizontal rectangles, which is in accordance with the theory that attention may be allocated more easily along the horizontal meridian. The fact that so few individuals exhibit a significant object-based cueing effect presumably is why previous studies of this effect might have yielded inconsistent results. The results from the current study highlight the importance of considering individual subject data in addition to commonly used statistical methods.

## Introduction

Visual attention can be allocated advantageously to locations in space (space-based attention; e.g., [1]) and to objects (object-based attention; e.g., [2]–[4]). Space-based attention is a process that allocates attention to a specific region, or location(s), in the visual field, whereas object-based attention directs attention to coherent forms or objects in visual space. In object-based attention, all parts of the attended object are thought to be processed concurrently. Consequently, the processing of parts within a single attended object is faster than processing parts across separate objects. Although space- and object-based attention can be influenced by top-down processes [5], [6] they also are thought to act in a bottom-up manner, and in concert with one another, to facilitate automatic processing of the visual world [4]. Consistent with this idea, space-based effects usually are very large and robust. However, object-based effects generally are much smaller and more variable in size. In fact, some studies have failed to find significant object-based cueing effects [7]–[13].

To address the issue of increased variability in object-based attention, the current study employed three common object-based attention paradigms to investigate the prevalence of object-based attention in large groups of subjects, and to determine the extent to which object-based effects vary across tasks [2]–[4].

One of the most widely used paradigms to study object-based attention was introduced by Egly et al. [2]. In this task, two parallel rectangles appeared on a uniform background, and, after a short delay, a cue was briefly presented by brightening a portion of the outline of one rectangle's end. The cue then disappeared, and after a short interval a target appeared on the screen. The subject's task was to respond to the onset of the target, which was a filling-in of an end of one of the rectangles. Subjects responded faster to targets that appeared at the cued end of the rectangle (Valid trials) than to targets at non-cued locations (Invalid trials), which is in accordance with theories of space-based attention. However, because Egly et al. included two objects in their displays, targets on Invalid trials could appear either on the opposite end of the cued rectangle (Invalid-same trials) or at the same end of the non-cued rectangle (Invalid-different trials). Critically, the spatial separation between the cue and target was the same in both kinds of Invalid trials. Interestingly, subjects responded faster on Invalid-same trials than Invalid-different trials. In other words, subjects exhibited an object-based attention effect.

Another paradigm to study object-based attention was introduced by Moore et al. [3]. Whereas the Egly task required target detection, Moore et al. modified the task in a way that subjects had to discriminate target letters. On each trial, four characters appeared at the ends of the two rectangles after the cue had disappeared. Targets were either the letter T or the letter L, and distractor items were T-L hybrid characters. Subjects had to identify the target letter presented. Although error rates and reaction times were generally higher in this task, overall, the results remained the same as already described by Egly et al. [2]. Several studies have replicated Moore et al.'s results using similar discrimination paradigms (e.g., [14], [15]).

A third method of assessing the effects of object-based attention was used by Watson et al. [4]. Unlike the methods described above, the task used by Watson et al. did not rely on a cued shift of attention. Instead, subjects attended to object properties that were distributed either within a single object or across two objects. In their standard task, two wrench-like stimuli were presented very briefly on either side of a fixation cross. Each wrench could have one end that was bent, one end that contained a gap, or both kinds of ends. The subject's task was to determine whether one or two of the target properties were present in the briefly presented display. When two properties were present, response times were faster when both properties were part of the same wrench than when they were part of separate wrenches. Similar to the conclusions of Egly et al. [2], this pattern of results was interpreted as evidence of object-based attention.

All three tasks described above yield large space-based cueing effects, but rather small object-based cueing effects.

Egly et al. reported that the response time difference between trials with Valid and Invalid location cues was 40 ms, whereas the difference between trials on which parts of the same or different objects were cued was only 13 ms. Similarly, Hecht and Vecera [16], using a discrimination task, found a space-based cueing advantage of 115 ms, but an object-based effect of only 19 ms. Many other researchers have reported similar results using both target detection and discrimination, with object-based effects typically being 2–4 times smaller than space-based effects (e.g., [3], [10], [15], [17]–[20]). Furthermore, several studies have failed to find significant object-based attention effects, even though their methods and stimulus conditions were similar to studies that found the effect. For example, [12] failed to find significant object-based effects using a paradigm that was similar to the one used by Egly et al. [2], but presented endogenous cues at fixation instead of exogenous cues at the possible target locations in the periphery. Similarly, [7] failed to find a significant object-based effect using rectangles that had been curved to resemble ribbons. Both studies did, however, obtain significant space-based cueing effects. Using a paradigm similar to [4], in which subjects had to respond to two target features in a display, [9] and [8] also failed to find object-based attention effects. These studies suggest that object-based attention is influenced strongly by cue type (i.e., exogenous versus endogenous [12], [17]), the stimulus-onset asynchrony (SOA) between cue and target [7], [11], [17], [21], [22], and the predictability of the cue location [21], [23]–[25].

These constraints on object-based cueing effects, and the relatively small size of object-based attention effects compared to the size and prevalence of space-based attention effects, raise questions about the reliability and/or prevalence of object-based attention. Therefore, in three experiments, using the paradigms by Egly et al. [2], Moore et al. [3], and Watson et al. [4], we investigated the robustness of object-based attention effects at the level of individual subjects. The current study took a novel approach to analyzing data collected in traditional object-based attention paradigms: in addition to commonly-used parametric statistical methods to compare performance across conditions and groups (e.g., analyses of variance (ANOVA), t-tests, etc.), we also used non-parametric bootstrapping procedures (e.g.,[26]–[28]) to evaluate the object-based attention effect at the level of individual subjects.

## Methods

### Experiment 1

#### Subjects

Sixty undergraduate students from McMaster University, Ontario, Canada (M = 20.01 years; 22 males) participated in this experiment. All subjects had normal or corrected-to-normal visual acuity, and were compensated for their time with partial course credit or $10/hour. The study was approved by the McMaster University Research Ethics Board and all subjects of this and the following experiments gave written informed consent.

#### Stimuli and Procedure

All stimuli were presented on a 19-in Sony GDM-C520 monitor, with a refresh rate of 75 Hz and a resolution of 1152
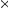
870 pixelss (39.5 cm
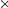
29 cm). Stimuli were generated and presented on a Macintosh G5 (OS X) Apple computer using Matlab (v 7.0) and the Psychophysics and Video Toolboxes [29], [30]. Subjects viewed the stimuli binocularly at a distance of 57 cm while seated in an adjustable chair in a darkened room. A chin rest was used to stabilize head position and viewing distance throughout the experiment.

**Figure 1:**
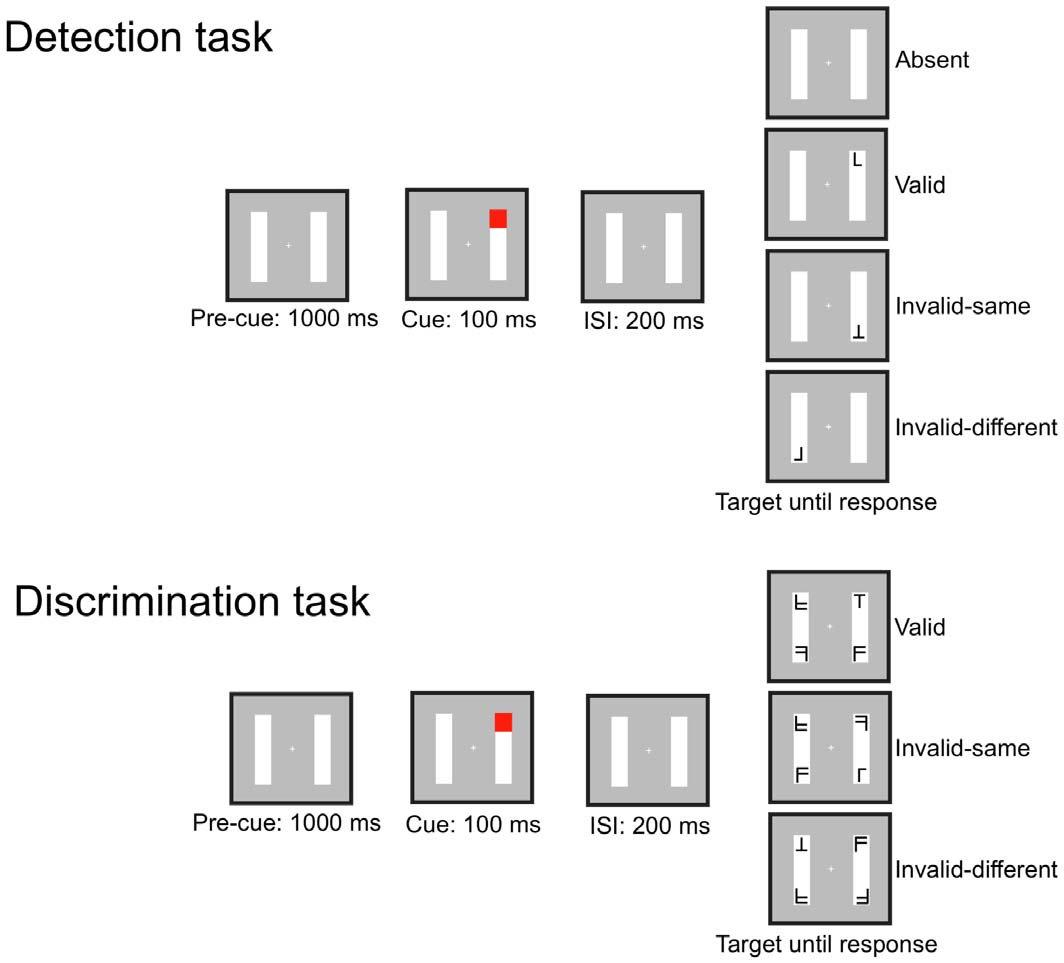
Schematic diagram of single trials in Experiment 1 for the detection task (top panel) and the discrimination task (bottom panel).

Stimulus displays were composed of two white rectangles (each 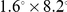) with a luminance of 91.2 cd/m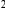, and a white fixation cross (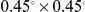) located in the center of the screen (see Figure 1). The two rectangles were either oriented vertically and located on either side of the fixation cross, or oriented horizontally and located above and below the fixation cross. The inner edges of the two rectangles were separated by 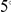. The fixation cross was located 2.5 from the inner edge of each rectangle.

The two rectangles were presented for 1 s on the screen at the start of the trial. A 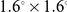 square cue was then presented, which consisted of one end of one of the rectangles briefly being filled in red (luminance: 13.4 cd/m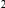; CIE: 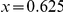, 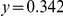).

The cue was presented for 100 ms, and the onset of the target display occurred 200 ms after the offset of the cue. Targets were either the letter T or the letter L, presented with equal probability in an upright orientation, or rotated 90, 180, or 270. The lines comprising the targets subtended a width of 0.05, and the entire letter measured 0.6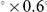.

Finally, a target could appear at the end of the rectangle where the preceding cue had appeared (Valid trials), or in one of the two possible uncued locations that were equally far apart from the cued location (Invalid trials). Targets stayed on the screen until the subject made a response. Subjects were told to respond as accurately and quickly as possible.

Subjects participated in two experimental sessions. In one session, they performed a detection task as in Egly et al., in which they had to respond as soon as they saw the target appear. Each subject was shown 640 target-present trials consisting of 480 (2 object orientations
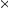
4 target locations
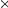
60 repetitions) Valid trials (75%), in which the target appeared at the cued location, and 160 (2 object orientations
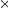
2 invalid conditions) Invalid trials, in which the target appeared at an uncued location that either was or was not on the same object as the cued location. On Invalid-Same trials, the target appeared on the same object as the cue but at the opposite end of the rectangle. On Invalid-Different trials, the target appeared on the opposite object at the end that was closest to the cue. The distance between the cue and the target was the same on Invalid-Same and Invalid-Different trials.

Finally, there were also 128 catch trials (16
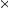
2 object orientations
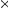
4 cue locations) in which the cue was not followed by a target. Subjects had to press the space bar as quickly and accurately as possible whenever a target appeared at one of the four rectangle ends and to withhold responses on the trials in which no target appeared. Each trial was terminated when a response was made or after 2000 ms in case of no response. There were eight blocks of 96 trials in this session, and the trial order was randomized across all eight blocks. An illustration of the experimental paradigm is shown in Figure 1 (top panel).

In the other session, subjects performed a discrimination task as in Moore et al. [3], in which they had to determine the identity of the target. The discrimination task used the same cue as the detection task. In the discrimination task, however, the cue was followed by the presentation of four characters at the ends of the two rectangles.

Target items were either the letter T or the letter L as described above, and distractor items were T-L hybrid characters, which were the same size, and had the same line width as the target items. Following Moore et al. [3], on each trial one of the target items (T or L) was always present, and the other three ends of the rectangles were filled with a distractor item. Each subject performed 240 trials: as was done by Moore et al. [3], 192 (80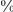) were Valid trials, in which the target appeared at the exact same position as the cue, 24 (10%) were Invalid-Same trials, and 24 trials Invalid-Different trials.

As was the case in the detection task, the distance between the cue and target was the same on the Invalid-Same and Invalid-Different trials. Subjects were asked to identify the target letter presented as quickly and accurately as possible by pressing the “/” key (for T's) or the “z” key (for L's) on a QWERTY keyboard. An illustration of the experimental paradigm is shown in Figure 1 (bottom panel).

Each session was preceded by 10 practice trials to familiarize subjects with the procedure.The order of detection and discrimination tasks was randomized across subjects.

#### Analysis

An analysis of variance (ANOVA) assessed the effects of the cue at the three possible target locations on both reaction time (RT) and error rate. An arcsin transform was used to normalize the distribution of the accuracy data [31]. A repeated measures design (Orientation (Vertical, Horizontal)
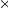
Cueing condition (Valid, Invalid-same, Invalid-different)) ANOVA evaluated the impact of orientation and cue validity on performance. Planned one-tailed t-tests also were carried out to evaluate both the general effect of cueing the target location, as well as whether there was an object-based effect of cueing. Space-based cueing effects were calculated for each subject by subtracting error rate or RT data from Valid trials from error rate or RT data on Invalid trials. Object-based cueing effects were calculated for each subject by subtracting the error rate or RT data on Invalid-Same trials from the error rate or RT data on Invalid-Different trials. As described in [3] and [2] RTs of less than 150 ms were excluded as anticipatory responses, and false-alarm RTs were not analyzed.

In addition to the ANOVA, the percentile bootstrap method [26]–[28] was used to estimate confidence intervals for space- and object-based cueing effects for individual subjects. This was done as follows: For each subject, 999 bootstrapped data sets were constructed by resampling the original data randomly with replacement. The data in each condition of the bootstrapped data sets were resampled with replacement from the corresponding condition in the original data set so that the bootstrapped data sets contained the same number of trials in each condition as the original data. Each bootstrapped data set was analyzed like the original sample: Mean RTs for space- and object-based cueing effects were calculated for RTs larger than 150 ms and correct responses. Bootstrapped space-based cueing effects were calculated by comparing the bootstrapped means on Valid trials and all Invalid trials (i.e., the combination of Invalid-same and Invalid-different trials). Bootstrapped object-based cueing effects were calculated by comparing the means of Invalid-same and Invalid-different trials. The 2.5 and 97.5 percentiles of the 999 bootstrapped cueing effects were then used to estimate the 95% confidence intervals for the space- and object-based effects for each subject.

Finally, to simulate the results of object-based attention experiments using sample sizes more typically collected in experiments of these types [2], [3], [7], [17], [21], [22], we randomly sampled 16 subjects from the 60 subjects 999 times, and performed one-tailed t-tests on each group to determine the number of times that the difference was significant in the direction predicted by theories of object-based attention (Invalid-same RT
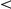
Invalid-different RT) and space-based attention (Valid RT
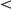
Invalid RT) for each orientation condition.

### Experiment 2

#### Subjects

One hundred and twenty undergraduate students from McMaster University, Ontario, Canada (M = 20.5 years; 34 males) participated in this experiment. All subjects had normal or corrected-to-normal visual acuity and were compensated for their time with partial course credit.

#### Stimuli and Procedure

Stimuli and procedure were similar to the ones used in the discrimination task in Experiment 1. This time, however, subjects completed more trials and the orientation of the rectangles was varied between subjects: each subject performed the task on either horizontal or vertical rectangles. In addition, the cue was not a filling in of one end of the rectangles but just the presentation of a partial outline at the end of a rectangle.

Subjects completed two sessions. During the first experimental session, subjects became accustomed to the task by completing 10 practice trials. Within each experimental session, subjects completed 336 Valid trials, 56 Invalid-same trials, and 56 Invalid-different trials, for a total of 896 trials in both sessions.

#### Analysis

The analysis was similar to the one used in Experiment 1: An analysis of variance (ANOVA) assessed the effects of the cue at the three possible target locations on both reaction time (RT) and error rate. Space- and object-based effects were calculated as in Experiment 1. A between-within design ANOVA evaluated the impact of stimulus orientation (Vertical and Horizontal) and cue validity (Valid, Invalid-same, Invalid-different) on performance. Planned one-tailed t-tests also were carried out to evaluate both the general effect of cueing the target location, as well as whether there was an object-based effect of cueing. RTs of less than 150 ms were excluded as anticipatory responses, and false-alarm RTs were not analyzed. In the current study, 24 trials for one and 73 trials for another subject had to be excluded for further analysis due to RTs of less than 150 ms.

In addition to the ANOVA, a percentile bootstrap method was used to estimate confidence intervals for space- and object-based cueing effects for individual subjects, similar to Experiment 1. Because confidence intervals for space-based effects did not vary much between the two methods used to compute space-based effects for the discrimination task in Experiment 1, here we only used the common method to calculate space-based confidence intervals for individual subjects by including both Invalid-same and Invalid-different trials. In addition 16 subjects were randomly sampled from the 60 subjects in each group 999 times and differences between Invalid-same and Invalid-different trials were compared for each orientation condition.

### Experiment 3

#### Subjects

Sixty undergraduate students from McMaster University (M = 20.2; 15 males) participated in this experiment for $15 or partial course credit. All had normal or corrected-to-normal visual acuity, and none had participated in Experiments 1 or 2.

#### Stimuli and Procedure

Stimuli were presented on a 19-in. monitor with a resolution of 1152
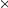
870 pixels (39.5 cm
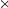
29 cm) and a refresh rate of 75 Hz. Stimuli were generated and presented on a Macintosh G5/350 (OS. 9.2.2) Apple computer using Matlab (v 5.2) and the Video and Psychophysics Toolboxes (Brainard, 1997; Pelli, 1997). The “D” and “L” keys on a standard QWERTY keyboard were used to make responses, as these were the keys used in the original [4] study. Subjects viewed the stimuli binocularly from a distance of 62 cm while seated in an adjustable chair in a well-lit room. A chin rest stabilized head position throughout the experiment.

**Figure 2:**
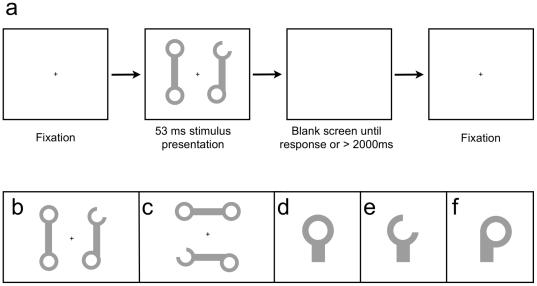
a) Example of a single trial in Experiment 3; b) Example of vertically oriented wrench display; c) Horizontally oriented wrench display; d) Intact wrench end; e) Wrench end with gap; and f) Bent wrench end.

The stimuli were wrench-like objects, drawn to the specifications listed by Watson et al. [4] (see Figure 2). Each display contained two wrench-like objects, the ends of which were designated as target or non-target ends. Target wrenches contained one or two properties: a bend at one end, a gap at one end, or a bend on one end and a gap on the other end. Non-target wrench ends consisted of an enclosed circle. The distance between target properties, measured from the exterior edge of one wrench end to the exterior edge of the other end, was held constant within and between wrenches.

The wrenches were not outlined: they were uniform grey (38.6 cd/m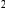) figures presented on a white (69 cd/m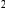) background. All wrench ends had a diameter and thickness subtending 1.8 and 0.5 respectively, and were located approximately 3.0 from a central black fixation cross (subtending 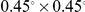). The wrench shaft measured 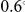 in width. A 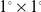 square was used to create the appearance of a gap by removing the quadrant of that wrench end furthest from fixation. The appearance of a bend was created by displacing one of the wrench ends 0.6, both vertically and horizontally, closer to the fixation point. The entire wrench display subtended a visual angle of approximately 6.0 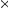
6.0 when neither end was bent, with a distance of 2.4 separating target properties. These measurements were reduced to 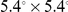 and 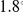 respectively when a bent end appeared in the display.

On each trial, subjects pressed a key to indicate whether they had seen one (bend or gap) or two (bend and gap) target properties in that particular wrench display. There was an equal probability that the wrench display contained one or two target properties. For trials that included only one target property, each end-type (the bend or gap) was equally likely to appear. When two properties were present, they could be part of one or two objects, but, to maintain an equal distance between properties, they could never appear diagonally from each other.

The response keys were labeled “1” or “2” to indicate the appropriate response; half of the subjects responded 1 with their left hand and half made that response with their right hand. On each trial, wrenches could be presented, with equal probability, at either a horizontal or vertical orientation. In the horizontal configuration, one wrench was located above and the other below the fixation cross; in the vertical configurations, one wrench was located to the left and the other to the right of the fixation cross. Subjects received auditory feedback (a low-pitched tone) immediately after each trial for which there was an incorrect response. At the end of each block, subjects were shown their percentage of correct responses for that particular block and the percentage for their overall performance. Text displayed on-screen reminded subjects to try to maintain accuracy above 90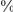 while still responding as quickly as possible. All subjects were tested with horizontal and vertical stimuli. The orientation of the wrenches was randomized across trials for each subject individually.

At the start of the experimental session, subjects were given a verbal explanation of the experimental task and then guided through several practice trials to ensure the instructions were understood. Each block of practice trials consisted of eight trials in which the wrenches remained visible on-screen until the subject made a response. The subject had to correctly respond to all eight trials in a block before the experimental trials would begin. As many practice blocks as necessary were given until this criterion was met. Subjects typically required no more than one practice block; the maximum needed by any subject was five.

The experiment consisted of 18 blocks of 64 trials each (1152 trials in total). Subjects were encouraged to take a self-timed break after every six blocks. After fixating a central fixation cross, subjects pressed the space bar to start a trial and, after a delay of 1000 ms, the wrenches were briefly flashed for 53 ms. The display then remained blank until either the subject responded or exceeded a time limit of 2000 ms. Exceeding this time limit resulted in a low tone, and the trial was counted as incorrect.

#### Analysis

Data were analysed as described in [4]: The analysis included all trials in which two stimulus properties were presented because these are the trials from which an object-based effect can arise. Only RTs for correct responses were analyzed. For each subject in each condition, RTs that were beyond 3 standard deviations from the mean were excluded from the analysis. Object-based effects were calculated by comparing trials in which the two stimulus properties were presented on the same wrench (same-object trials) with trials in which the two stimulus properties were presented on different wrenches (different-object trials).

Like in the previous experiments, a bootstrap method was used to estimate confidence intervals for object-based cueing effects for individual subjects. This was done as follows: For each subject, 999 bootstrapped data sets were constructed by resampling the original data randomly with replacement. Only trials in which two stimulus properties were presented were included. Each bootstrapped data set was analyzed like the original sample: Mean RTs object-based cueing effects were calculated for RTs less than 3 standard deviations from the mean. Bootstrapped object-based cueing effects were calculated by comparing the means of same-object and different-object trials. The 999 bootstrapped cueing effects were then used to estimate 95% confidence intervals for the space- and object-based effects for each subject.

To simulate the typical sample sizes for previous object-based attention studies using this paradigm, we randomly sampled 16 subjects from the 60 subjects 999 times, and performed one-tailed t-tests on each group to determine the number of times that the difference was significant in the direction predicted by theories of object-based attention (same-object RT
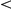
different-object RT).

## Results

### Experiment 1

#### Accuracy

**Figure 3:**
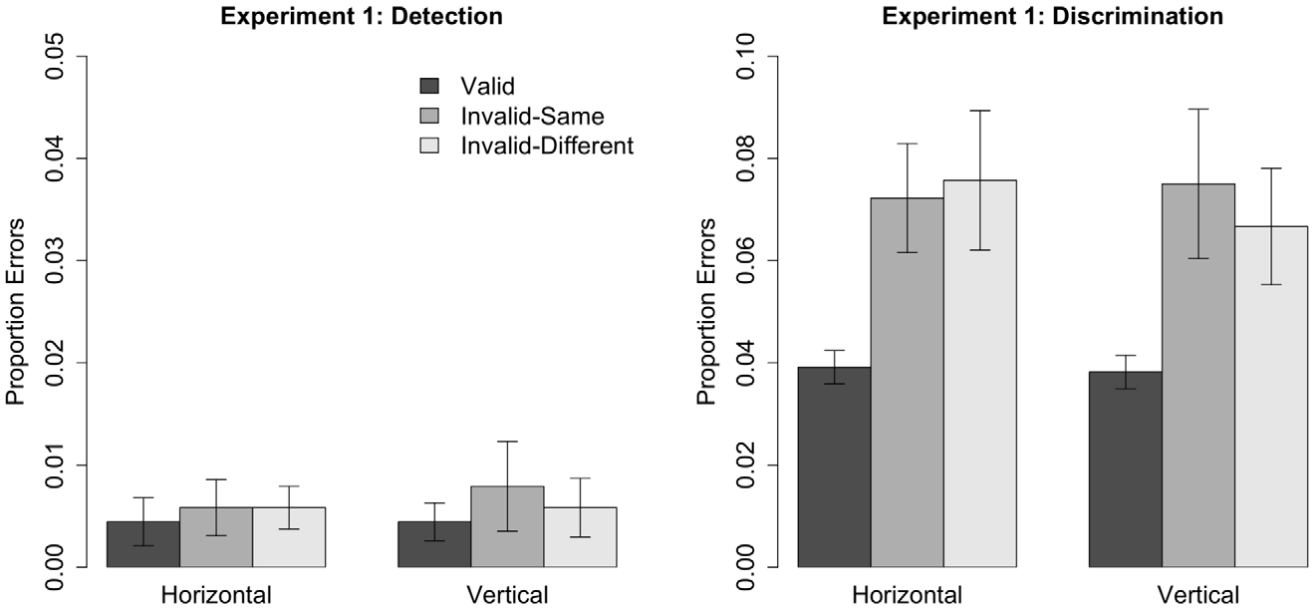
Mean error rates for the detection (left) and discrimination (right) tasks in Experiment 1. Error bars depict standard errors. Note that the ordinate differs in the two figures.

Figure 3 shows the error rates for both the discrimination and the detection task. A 2 (Task)
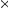
2 (Orientation)
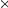
3 (Cue) within-subjects ANOVA on the arcsine-transformed error rates revealed a significant main effect of Task (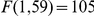, 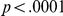). The main effects of Orientation (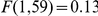, 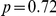) and Cue (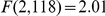, 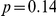), as well as the Task
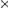
Cue interaction (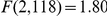, 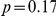), were not significant. All of the remaining interactions did not approach significance (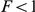 and 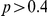 in all cases).

Space-based cueing effects were analyzed with a 2 (Task)
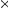
2 (Orientation) within-subjects ANOVA. The overall spaced-based cueing effect (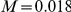, 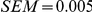) was significant (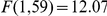, 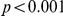). There was a significant main effect of Task (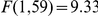, 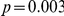), which reflected the fact that the space-based cueing effect was greater in the discrimination task (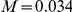, 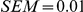) than the detection task (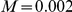, 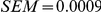). Despite their small magnitudes, space-based cueing effects were significantly greater than zero in both tasks (detection: 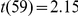, 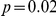; discrimination: 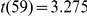, 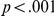). The main effect of Orientation (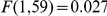, 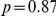) and the Task
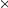
Orientation interaction (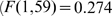, 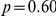) were not significant. In summary, there was a significant space-based cueing effect that varied between tasks but not between orientations.

Object-based cueing effects were analyzed with a 2 (Task)
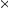
2 (Orientation) within-subjects ANOVA. The overall mean object-based cueing effect (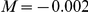, 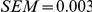) did not differ from zero (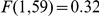, 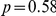). Furthermore, the main effects of Task and Orientation, and the Task
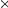
Orientation interaction, were not significant (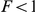 and 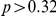 in all cases). Hence, there was no evidence of an object-based cueing effect on response accuracy in either task at either orientation.

#### Reaction Times

As was done by Moore et al. [3] and Egly et al. [2], only RTs for correct responses were analyzed. Furthermore, RTs of less than 150 ms were classified as anticipatory responses and excluded from further analyses. It turns out, however, that there were no anticipatory responses in the current experiment. For the detection task we also analysed catch trials to investigate whether the number of false alarms might have influenced the results. Subjects responses on average were highly accurate (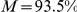, 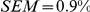) and there were no RTs less than 150 ms. Hence, the responses on catch trials suggest that subjects did not make a large number of anticipatory responses.

**Figure 4:**
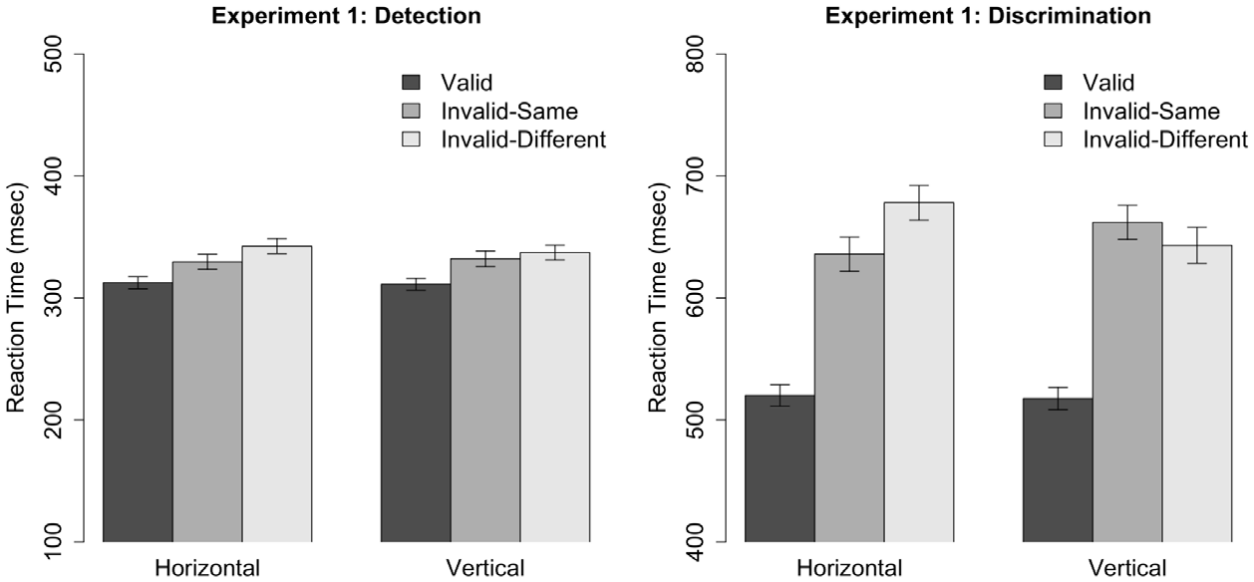
Mean reaction times (RT) for the detection (left) and discrimination (right) tasks in Experiment 1. Error bars depict standard errors. Note that the ordinate differs in the two figures.

Figure 4 shows RT data for both tasks. A 2 (Task)
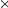
2 (Orientation)
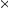
3 (Cueing condition) within-subjects ANOVA found main effects of Task (F(1,59) = 812; 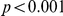) and Cueing condition (F(2,118) = 213; 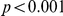). There were significant interactions between Task and Condition (F(2,118) = 124, 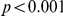), Orientation and Cueing condition (F(2,118) = 23.5, 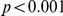) and Task, Orientation and Cueing condition (F(2,118) = 15, 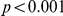). The main effect of Orientation (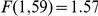, 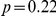) and the Task
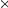
Orientation interaction (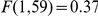, 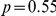) were not significant.

Space-based cueing effects were analyzed with a 2 (Task)
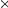
2 (Orientation) within-subjects ANOVA (see Table 1). There was a significant effect of Task (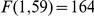, 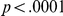), which reflected the fact that the space-based cueing effect was greater in the discrimination task than the detection task. Nevertheless, the cueing effect was significant in both tasks (detection: 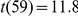, 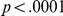; discrimination: 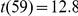, 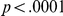). The main effect of Orientation (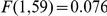, 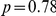) and the Task
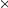
Orientation interaction (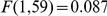, 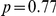) were not significant, hence space-based cueing effects did not vary with object orientation in either task.

**Table 1:** Mean space-based RT cueing effects (msec) in Experiment 1.

	Detection	Discrimination	Mean
Horizontal	23.5 (2.2)	136.8 (9.4)	80.1 (7.1)
Vertical	23.5 (2.3)	134.9 (9.5)	79.2 (7.0)
Mean	23.5 (1.6)	135.8 (6.7)	79.7 (5.0)

Values in parentheses are standard errors.

Object-based cueing effects were analyzed with a 2 (Task)
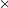
2 (Orientation) within-subjects ANOVA (see Table 2). The main effect of Task was not significant (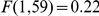, 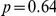), however the main effect of Orientation (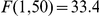, 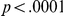) and the Task
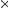
Orientation interaction (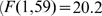, 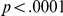) were significant. To decompose the interaction, object-based cueing effects in the detection and discrimination tasks were analyzed separately. In the detection task, the overall cueing effect was significant (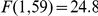, 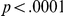), but the significant effect of Orientation (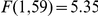, 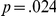) indicated that the cueing effect was larger for horizontal rectangles. Separate 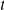 tests indicated the object-based cueing effect was significantly greater than zero at both orientations (horizontal: 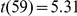, 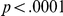; vertical: 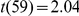, 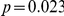). In the discrimination task, the overall cueing effect was significant (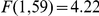, 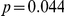), but, as was found in the detection task, the significant main effect of Orientation (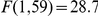, 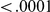) indicated that the cueing effect was larger for horizontal rectangles. 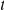 tests indicated that the cueing effects differed significantly from zero for both horizontal (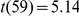, 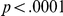) and vertical (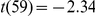, 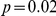) rectangles. Unlike what was found in the detection task, however, the effect obtained with vertical rectangles was opposite to the effect predicted by theories of object-based attention (see Table 2).

**Table 2:** Mean object-based RT cueing effects (msec) in Experiment 1.

	Detection	Discrimination	Mean
Horizontal	12.8 (2.4)	42.1 (8.2)	27.5 (4.5)
Vertical	5.1 (2.5)	−18.8 (7.9)	−6.9 (4.2)
Mean	9.0 (1.8)	11.7 (6.3)	10.3 (3.3)

Values in parentheses are standard errors.

In addition, to test for the robustness of the object-based effects between object orientations, we computed correlation coefficients for object-based effects measured with horizontal and vertical rectangles. The correlation was not significant in the detection task (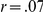, 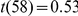, 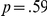) or in the discrimination task (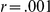, 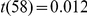, 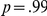).

#### Bootstrapping of individual subjects

**Figure 5:**
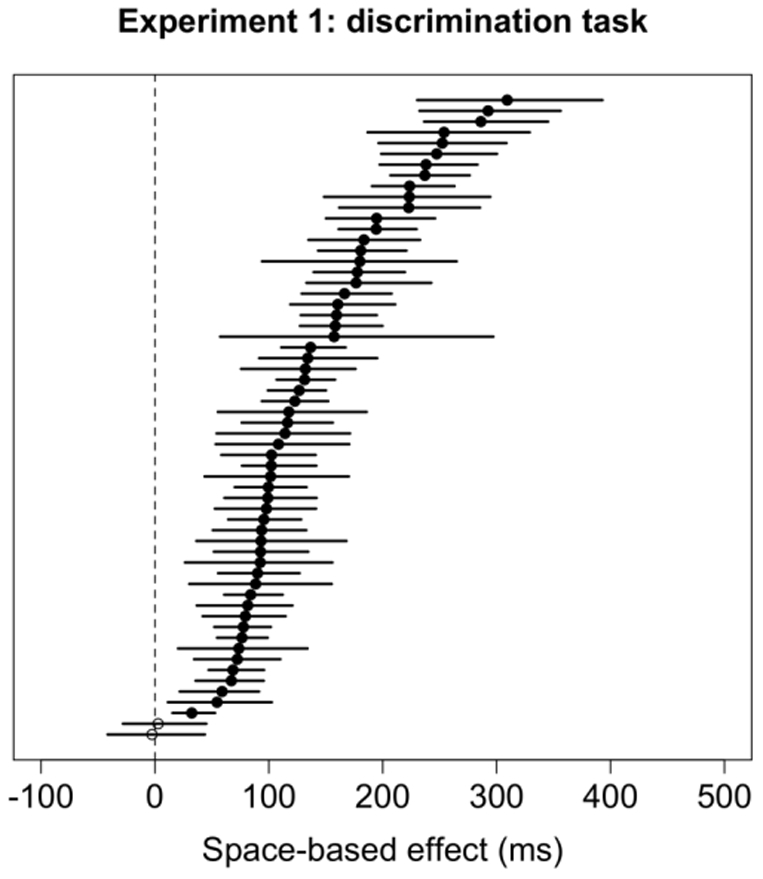
Spaced-based cueing effects (i.e., the difference between Valid and Invalid trials) and 95% confidence intervals for each subject in Experiment 1 for both vertical and horizontal objects in the discrimination task. Significant cueing effects are denoted by filled circles. The circles on the right side of the zero difference line denote a positive difference, which is in the direction predicted by theories of space-based attention.

**Figure 6:**
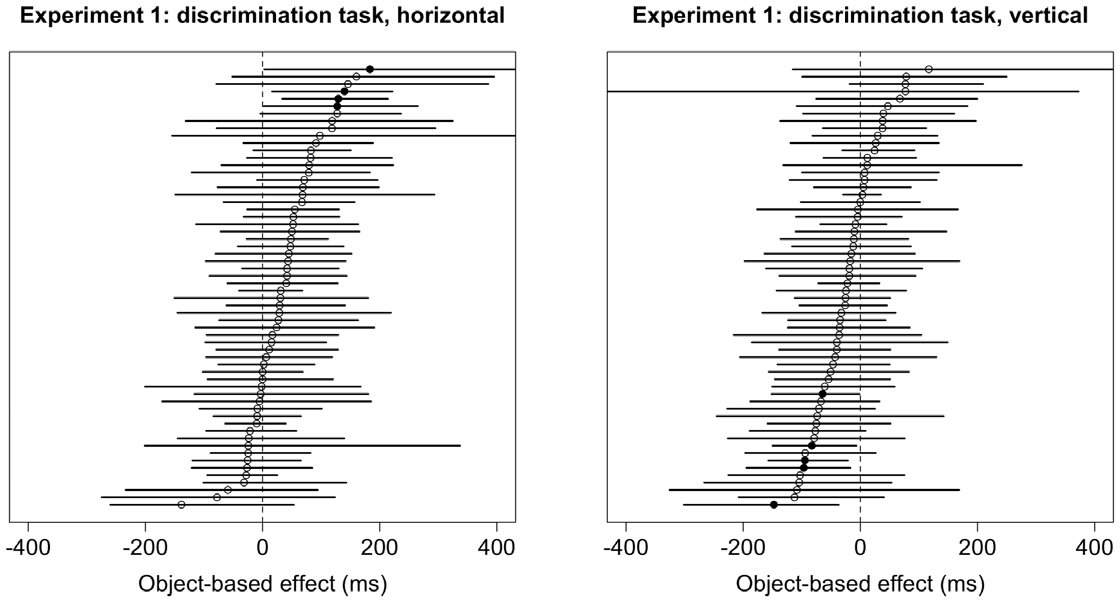
Object-based cueing effects (i.e., the difference between Invalid-same and Invalid-different trials) and 95% confidence intervals for each subject in Experiment 1 for horizontal (left) and vertical (right) objects in the discrimination task. Significant cueing effects are denoted by filled circles. The circles on the right side of the zero difference line denote a positive difference, which is in the direction predicted by theories of object-based attention.

Results of the bootstrap analysis for the space-based effect in the discrimination task are summarized in Figure 5. Results for individual subjects are significant when the 95% confidence interval does not cross 0. Across both orientations, 58 subjects (96.7%) showed significant space-based attention effects. Results for the object-based effect in the discrimination task vary much more across subjects, and are summarized in Figure 6 (results for horizontal rectangles on the left, and for vertical rectangles on the right). For horizontal rectangles, only 4 subjects (6.7%) exhibited a significant effect as predicted by object-based attention. For vertical rectangles, none of the subjects showed an effect as predicted by object-based attention, but 5 subjects (8.3%) showed an effect in the opposite direction (i.e., longer RTs for Invalid-same trials than for Invalid-different trials).

**Figure 7:**
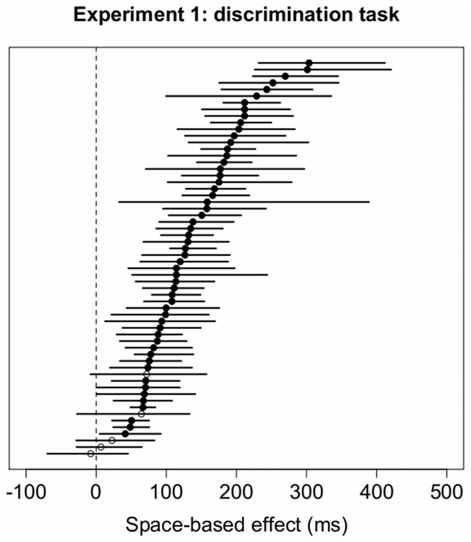
Space-based cueing effects as the difference between Invalid-same and Valid trials) and 95% confidence intervals for each subject in Experiment 1 for both vertical and horizontal objects in the discrimination task. The labeling conventions are the same as in Figure 5.

Commonly used procedures to compute space-based effects compare valid trials to both Invalid-same and Invalid-different trials (e.g., [2], [7]). This procedure confounds two distinct shifts of attention: Invalid-same trials contain only an attentional shift in space, while Invalid-different contain a shift in both space and object. To increase the compatibility of space- and object-based effects, we also decided to compute confidence intervals for space-based attention by comparing Valid trials to Invalid-same trials. The bootstrap procedure was the same as described above, only that bootstrapped space-based cueing effects were calculated comparing the means of Invalid-same and half the amount of Valid trials. When comparing space-based and object-based effects of attention independently of object, across both orientations, 56 subjects (93.3%) showed significant space-based attention effects as summarized in Figure 7.

**Figure 8:**
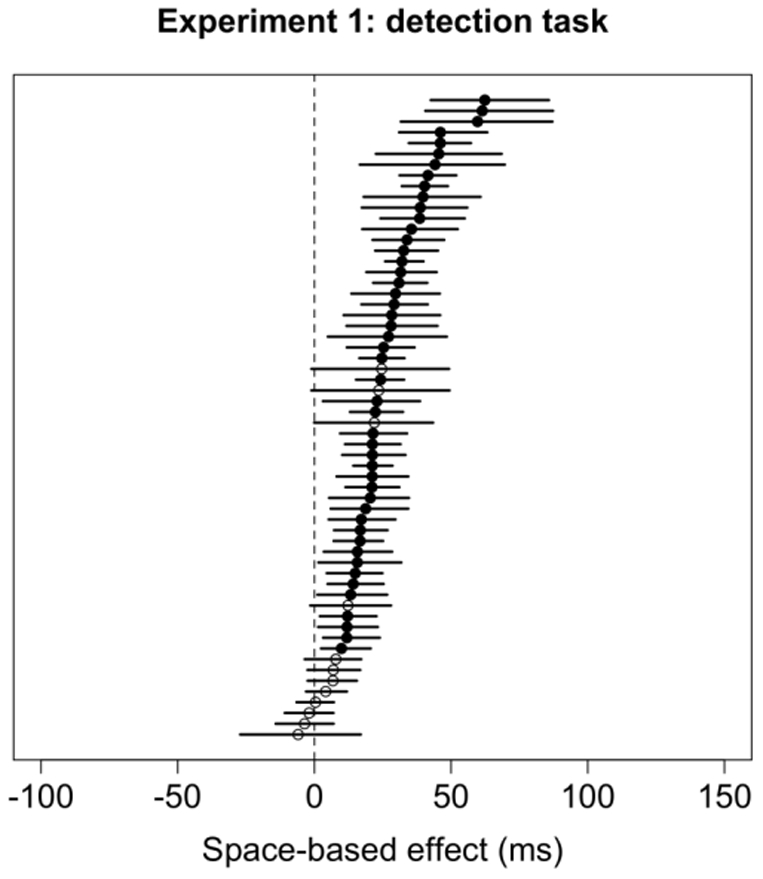
Spaced-based cueing effects (i.e., the difference between Valid and Invalid trials) and 95% confidence intervals for each subject in Experiment 1 for horizontal and vertical objects in the detection task. The labeling conventions are the same as in Figure 5.

**Figure 9:**
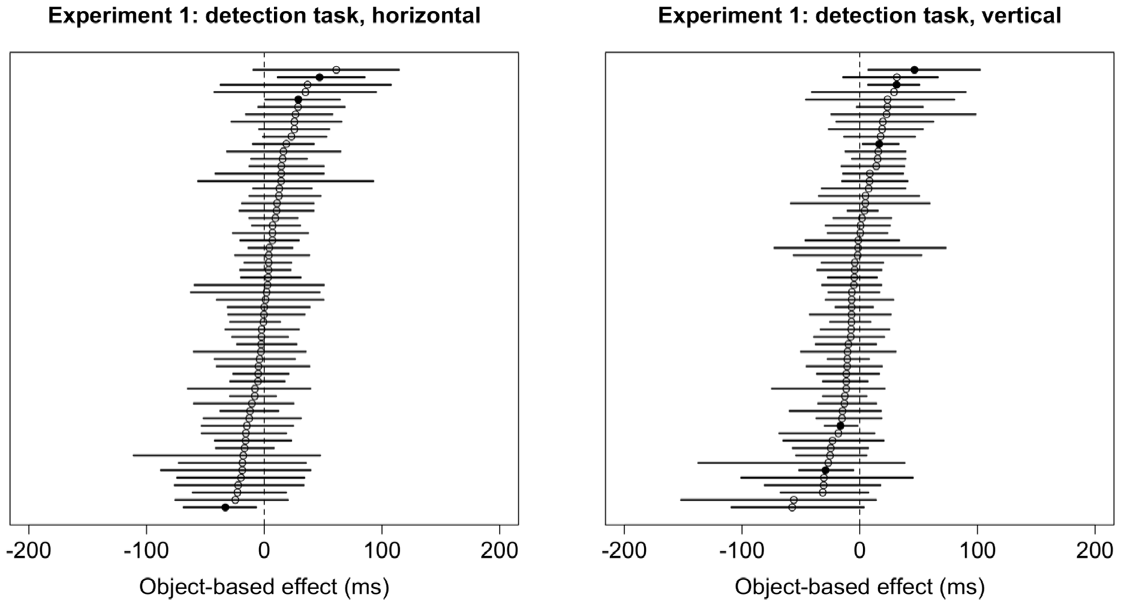
Object-based cueing effects (i.e., the difference between Invalid-same and Invalid-different trials) and 95% confidence intervals for each subject in Experiment 1 for horizontal (left) and vertical (right) objects in the detection task. The labeling conventions are the same as those in Figure 6.

**Figure 10:**
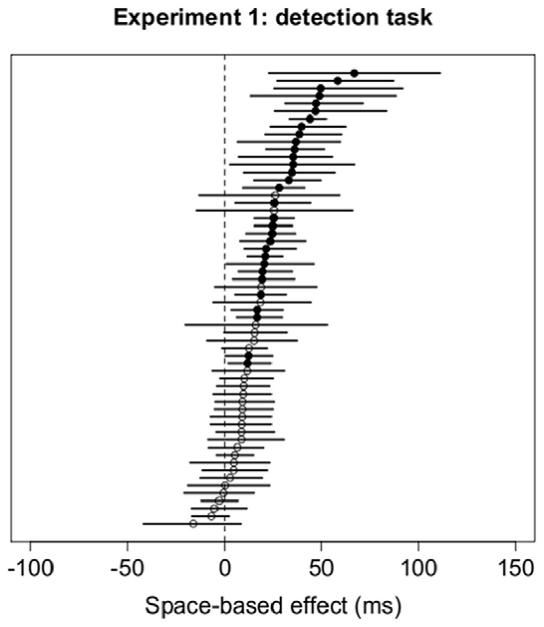
Space-based cueing effects as the difference between Invalid-same and Valid trials) and 95% confidence intervals for each subject in Experiment 1 for both vertical and horizontal objects in the detection task. The labeling conventions are the same as in Figure 5.

Results of the bootstrap analysis for the space-based and object-based effects in the detection task are summarized in Figures 8 and 9, respectively. Forty-nine subjects (81.7%) showed significant space-based attention effects. In contrast, significant object-based effects were seen much more rarely at the level of individual subjects: For horizontal rectangles, only two subjects (3.3%) showed a significant effect as predicted by object-based attention, and one subject showed a significant effect in the opposite direction; for vertical rectangles, three subjects (5%) showed a significant effect as predicted by object-based attention, and two subjects (3.3%) showed significant effects in the opposite direction. When comparing space-based and object-based effects of attention independently of object, across both orientations, 31 subjects (52%) showed significant space-based attention effects as summarized in Figure 10.

#### Random resampling of groups of subjects

The results of the random resampling are summarized in Table 3. For the discrimination task, *all* of the resampled groups (i.e., 999 or 100%) exhibited significant space-based cueing effects for both horizontal and vertical rectangles. However, only 80% of the resampled groups had significant object-based effects with horizontal rectangles, and only two resampled group had a significant object-based effect with vertical rectangles. Qualitatively similar results were obtained for the detection task, although in that task the probability of obtaining significant object-based cueing effects was higher than was the case for the discrimination task. These simulations suggest, therefore, that using the detection task increases the probability of obtaining significant object-based cueing effects when the sample size is 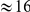.

**Table 3:** Results of resampling of groups of subjects (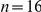).

Task	Cueing effect	Orientation	Groups	Percent	Effect (SD)
discrimination	space	both orientations	999	100%	136 ms (18)
	space	horizontal	999	100%	140 ms (18)
	space	vertical	999	100%	133 ms (20)
	object	both orientations	270	27%	23 ms (5)
	object	horizontal	803	80%	48 ms (13)
	object	vertical	2	0.2%	36 ms (5)
detection	space	both orientations	999	100%	24 ms (4)
	space	horizontal	999	100%	25 ms (4.5)
	space	vertical	999	100%	23 ms (4)
	object	both orientations	924	92%	10 ms (3)
	object	horizontal	916	92%	14 ms (4)
	object	vertical	297	30%	11 ms (3)

### Experiment 2

Experiment 1 showed that subjects performed generally better and faster at the detection task than the discrimination task. Although the space-based attention effect was large and reliable in both tasks, object-based attention effects were much smaller and more variable than those for space-based attention. When taking object orientation into account, small object-based effects were obtained for both rectangle orientations in the detection task, but the object-based attention effect in the discrimination task was significant in the predicted direction only for horizontal rectangles. The bootstrap analysis confirmed these results and showed that for both tasks and orientations the space-based effect was large and reliable, whereas the object-based effect was less reliable on the level of individual subjects and more pronounced for horizontal rectangles. The following experiment investigated the effect of object orientation in the discrimination task in further detail. We increased the number of subjects and the number of trials per subjects in each condition to get a larger number of trials to sample from and decrease the amount of inherent noise per subject. To achieve this, we tested orientation effects between subjects and also increased the number of subjects from 60 in Experiment 1 to 120 (60 per orientation condition).

#### Accuracy

**Figure 11:**
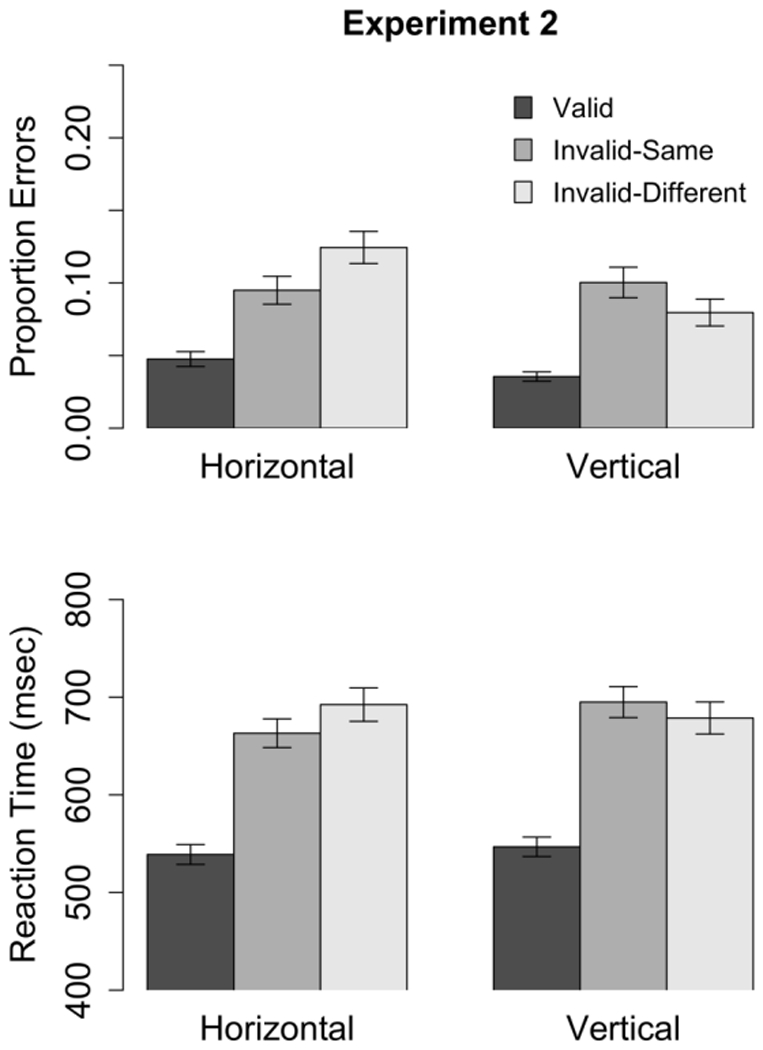
Mean error rate (top) and RTs (bottom) measured in Experiment 2 with horizontal and vertical rectangles. Error bars depict 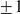 standard error.

Figure 11 (top) shows the error rates measured in each cueing condition. A 2 (Orientation)
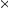
3 (Cue) mixed-design ANOVA on the arcsine-transformed error rates revealed significant main effects of Orientation (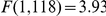, 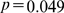) and Cue (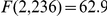, 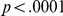), as well as a significant Orientation
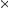
Cue interaction (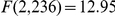, 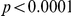). Inspection of Figure 11 suggests that the interaction reflects the fact that the error rate on Invalid-Different trials was higher with horizontal rectangles than with vertical rectangles.

Space-based cueing effects measured at different orientations were analyzed with a one-way, between-subjects ANOVA. The overall space-based cueing effect (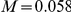, 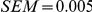) differed significantly from zero (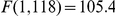, 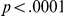) and the main effect of Orientation was not significant (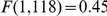, 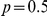). Hence, there was a significant space-based cueing effect that did not vary between object orientations.

Object-based cueing effects measured at each orientation were submitted to a one-way, between-subjects ANOVA. The overall object-based cueing effect (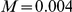, 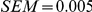) did not differ from zero (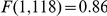, 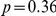). However, there was a significant main effect of orientation (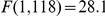, 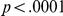), which indicated that the cueing effect depended on object orientation. The object-based cueing effects differed significantly from zero in both conditions (horizontal: 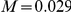, 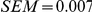, 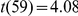, 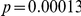; vertical: 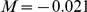, 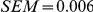, 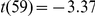, 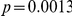), but the cueing effect obtained with vertical rectangles was in the direction opposite to that predicted by theories of object-based attention. In the vertical condition, in other words, subjects made *fewer* errors on Invalid-Different trials than on Invalid-Same trials (see Figure 11).

#### Reaction Times

As was done in Experiment 1, only RTs from correct trials that were longer than 150 ms were analyzed. In the current study, 24 trials for one subject and 73 trials for another subject had to be excluded for further analysis due to RTs of less than 150 ms. Figure 11 (bottom) shows mean RTs for the three cue conditions at each object orientation. Space-based cueing effects were analyzed with a one-way between-subjects (Orientation) ANOVA (see Table 4). The average space-based cueing effect differed significantly from zero (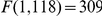, 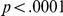) and the effect of orientation was not significant (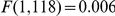, 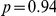), which indicated that the magnitude of space-based cueing effects did not vary with object orientation.

**Table 4:** Mean RT cueing effects (msec) measured in Experiment 2.

	Space-based	Object-based
Horizontal	138.8 (12.2)	29.2 (5.5)
Vertical	140.1 (10.2)	−16.3 (4.3)
Mean	139.4 (7.9)	6.5 (4.1)

Values in parentheses are standard errors.

Object-based cueing effects were analyzed with a one-way, between-subjects (Orientation) ANOVA (see Table 4). The average object-based cueing effect did not differ significantly from zero (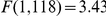, 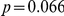). There was a significant main effect of orientation (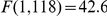, 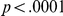), indicating that object-based cueing was larger for horizontal rectangles. Subsequent tests showed that that the object-based cueing effect differed significantly from zero at both object orientations (horizontal: 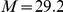, 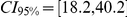 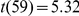, 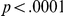; vertical: 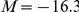, 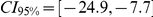, 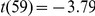, 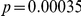). However, as was found in Experiment 1, the cueing effect obtained with vertical rectangles was in a direction opposite to the effect predicted by theories of object-based attention: with vertical rectangles, RTs were *faster* on Invalid-Different trials than on Invalid-Same trials (see Table 4). Note that this effect of orientation does not reflect a speed-accuracy trade-off because fewer errors were made on Invalid-Different trials with vertical rectangles (see Figure 11).

#### Bootstrap results

**Figure 12:**
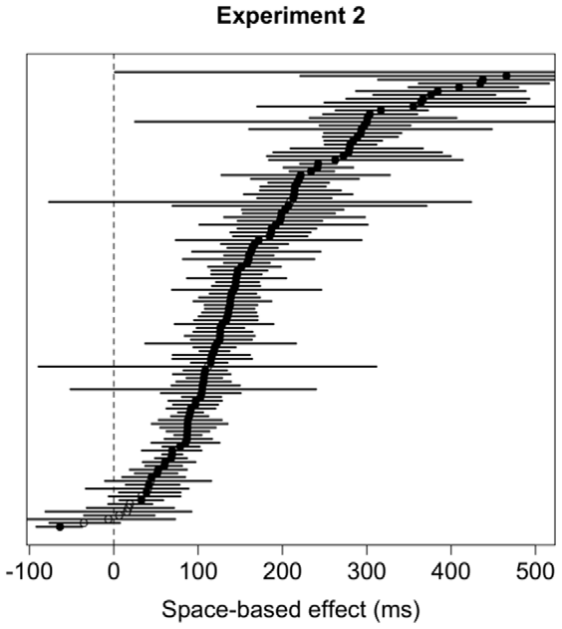
Distribution of mean differences between Validly-cued and Invalidly-cued trials and 95% confidence intervals for each subject in Experiment 2. Positive values are consistent with effects predicted by theories of space-based attention. Significant differences are denoted by filled circles; non-significant differences are shown with white circles.

**Figure 13:**
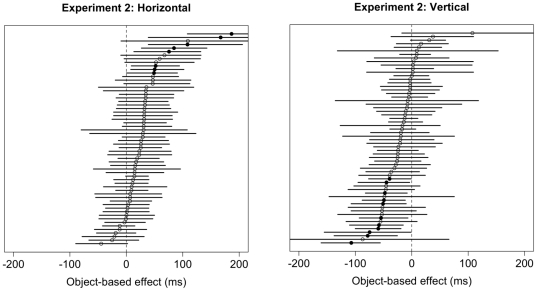
Distribution of mean differences between Invalid-different and Invalid-same trials and 95% confidence intervals for each subject in Experiment 2 for horizontal rectangles (left) and vertical rectangles (right). Positive values are consistent with effects predicted by theories of object-based attention. Significant differences are denoted by filled circles; non-significant differences are shown with white circles.

Results of the bootstrap analysis for the space-based effect are summarized in Figure 12. One hundred six (89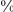) of the 120 subjects showed significantly faster responses for Valid trials, as predicted by theories of space-based attention. Only one (0.8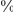) subject showed a difference in the opposite direction – i.e., faster responses for Invalid trials – and 13 (11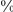) subjects showed no difference between Valid and Invalid trials. Results for the bootstrap analysis for the object-based effect for both horizontal and vertical rectangles are summarized in Figure 13. The bootstrap analysis for horizontal rectangles (Figure 13, left panel) revealed that only eight (13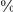) of the 60 subjects showed a significant cueing effect in the direction predicted by theories of object-based attention (i.e., faster responses for Invalid-same trials). The bootstrap analysis for vertical rectangles (Figure 13, right panel) revealed that only 11 (18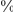) of the 60 subjects showed a significant cueing effect, all in the direction that is *opposite* to the one predicted by object-based attention.

#### Random resampling

For horizontal rectangles, 768 of the 999 resampled groups (i.e., 76.9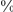) emerged with a significant difference in the predicted direction between the cue conditions (Mean difference = 29.5 ms, STD = 43.5 ms). For vertical rectangles, none of the groups showed an effect in the direction predicted by theories of object based attention (Mean difference = −16.3 ms, STD = 8.5 ms), but 985 (98.6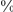) groups showed significant space-based effects for horizontal rectangles (Mean difference = 137.5 ms, STD = 83.5 ms). For vertical rectangles all groups showed significant space-based effects (Mean difference = 140 ms, STD = 18.7 ms).

### Experiment 3

Experiments 1 and 2 investigated the size and prevalence of space- and object based cueing effects using common detection and discrimination paradigms introduced by Egly et al. [2] and Moore et al. [3]. In both experiments, the size of the space-based cueing effect obtained in both the discrimination and the detection tasks was consistent with effects obtained in previous studies, and our bootstrap analyses indicated the effect was significant in nearly every subject. Significantly different results were obtained with object-based cueing effects, which, compared to space-based effects, were small and found only in a minority of subjects. Interestingly, object-based cueing effects were found more consistently with horizontal than vertical rectangles and, in the discrimination task used in both Experiments 1 and 2, subjects even showed an effect opposite to the one predicted by theories of object-based attention for vertically oriented stimuli.

A potential criticism of the discrimination and detection tasks used in the first two experiments is that object-based cueing effects might be smaller when the target is presented *on* an object rather than as *part of* an object. To evaluate this hypothesis, Experiment 3 used a different paradigm that was introduced by [4]. Most studies that have used this paradigm, though not all, have reported object-based attention effects that are larger than those typically reported in studies using Egly et al.'s (1994) paradigm, so we wondered whether the effects would be more robust in this paradigm at the individual subject basis as well.

#### Accuracy

**Figure 14:**
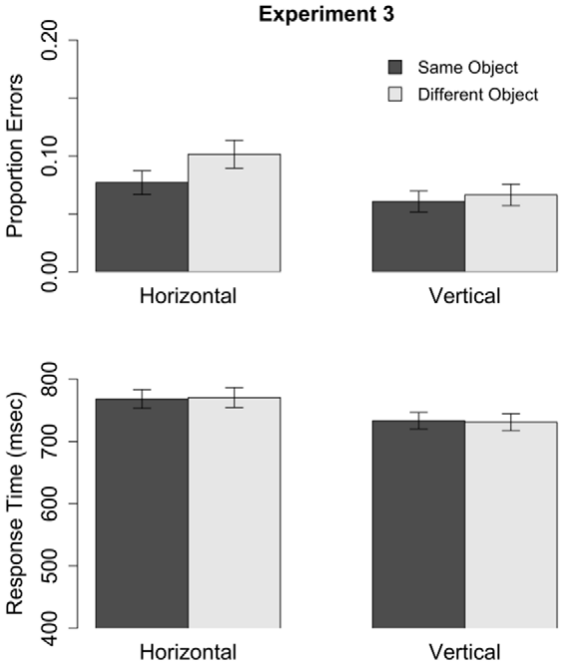
Mean error rate (top) and RTs (bottom) measured in Experiment 3 with horizontal and vertical rectangles. Error bars depict standard error.

The proportion of errors in each condition is shown in Figure 14. A 2 (Orientation) by 2 (Object) within-subjects ANOVA on arcsin-transformed error rates revealed a significant effect of stimulus orientation (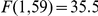, 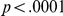): Subjects made significantly more errors with horizontal wrenches (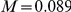, 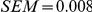) than with vertical wrenches (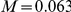, 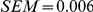). There also was a significant effect of Object (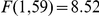, 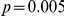): Significantly more errors were made on different-object (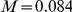, 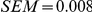) than same-object (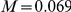, 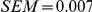) trials. The Orientation
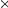
Object interaction approached, but did not reach, conventional levels of statistical significance (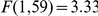, 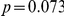).

Although the Orientation
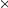
Object interaction was not significant, a subsequent ANOVA on the raw error rates (i.e., without using the arcsine transform) did find a significant interaction. In light of this result, and the results of the first two experiments, we thought it was reasonable to analyze the two orientations separately. Analyses of arcsine-transformed error rates indicated that the effect of Object was significant for trials using horizontal stimuli (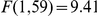, 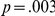) but not for trials using vertical stimuli (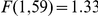, 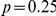).

#### Reaction times

Mean RTs are shown in Figure 14. There was a significant main effect of Orientation (F(1,59) = 62.73, 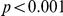): RTs for horizontal stimuli (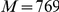, 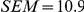) were slower than for vertical stimuli (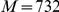, 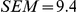). The main effect of Object (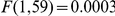, 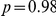) and the Object
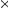
Orientation interaction (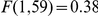, 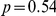) were not significant.

#### Bootstrapping

**Figure 15:**
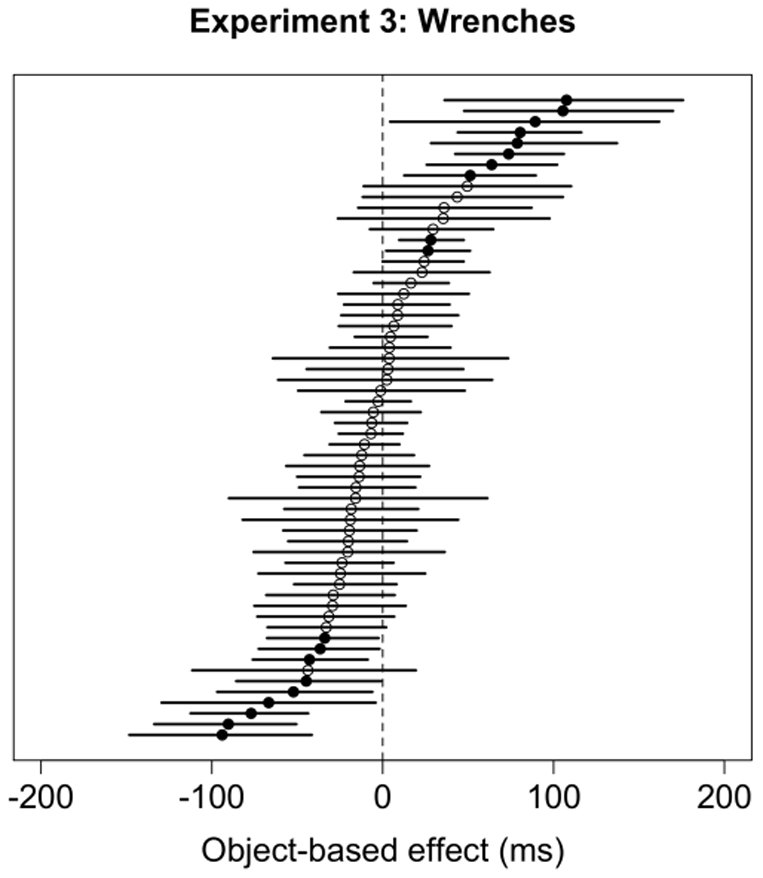
Distribution of mean differences between same-object and different-object trials, and 95% confidence intervals for each subject in Experiment 3. The difference between the two object conditions is significant if the 95% confidence interval does not include the value of 0. Significant differences are denoted by filled circles; non-significant differences are shown with white circles. The circles on the right side of the figure denote a positive difference, which is the opposite direction predicted by theories of object-based attention.

Because the Object
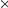
Orientation interaction in reaction times was not significant, we combined the data for both orientations to perform a bootstrap comparison of reaction time to targets presented on same- and different-object trials. The bootstrap comparison found a significant object-based attention effect (i.e., faster RTs on same-object trials) in only 10 subjects (7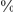; see Figure 15). Nine subjects (15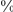) showed significant differences in the direction opposite from that predicted by object-based attention theories, and the remaining 41 subjects (68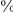) did not show a significant difference between same- and different-object trials.

#### Random resampling

We found that only 15 (1.5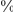) of 999 bootstrapped subject groups showed a significant difference between RTs in the same- vs. different-object conditions. Across all 999 samples, the difference between same-object vs. different-object trials ranged from −26 ms to 24.6 ms, with a mean and standard deviation of −1.75 and 8.5 ms, respectively.

## Discussion

In three experiments we evaluated the prevalence of space- and object-based attention at the level of individual subjects. Experiments 1 and 2 used slight variations of the target detection and target discrimination paradigms described by Egly et al. [2] and Moore et al. [3]. Experiment 3 employed a paradigm similar to the one used by [4]. Using common statistical analyses such as ANOVAs and t-tests, we found large and robust space-based attention effects, whereas object-based effects were small (Experiments 1 and 2, detection and discrimination task, horizontal rectangles), absent (Experiment 1, vertical rectangles, detection task; Experiment 3), or even inverted (Experiments 1 and 2, discrimination task, vertical rectangles). Bootstrapping showed that object-based attention appeared only in a small minority of subjects. In addition, we randomly resampled groups of subjects in each experiment and only a minority of groups showed significant object-based attention effects in all three experiments.

It has already been shown before that space-based attention effects are much larger and more robust to stimulus manipulations than object-based attention effects (e.g., [2], [3], [7], [10], [12], [16], [22]). Whereas space-based attention survives a variety of changes in the timing of cue and target, the object chosen, or the experimental task, object-based attention is much more vulnerable. But what is the reason for the qualitative differences between space- and object based attention and the low prevalence of object-based attention effects in single subjects as shown in the current study?

Some previous studies suggested that object-based attention effects are primarily based on bottom-up or image-based mechanisms [4], whereas other studies supported the hypothesis that top-down strategies are more important for object-based attention effects [6], [32]. The results from the current study suggest that processes that give rise to object-based cueing effects may occur only in a minority of subjects. Although it is possible that a bottom-up, image-based mechanism might operate only in a minority of subjects, we feel that our findings are more compatible with the idea that object-based effects reflect the influence of strategies that are adopted by a subset of subjects to perform the experimental task. Why would so few subjects adopt a strategy that produces object-based cueing effects? Although it might be advantageous in some situations to allocate attention to objects rather than locations, it is important to note that in the three paradigms used in our experiments there is no strategic advantage for the subject to choose to attend to an object as a whole. Just as the space-based attention effect can be manipulated by the predictability of the cue (e.g., [33]), it stands to reason that the probability that a subject uses the spatial arrangement of objects to guide the allocation of attention would be influenced by expectation or strategy. In other words, object-based effects may be found in more subjects if the tasks were changed to provide more incentive for subjects to attend to entire objects.

Indeed, the current study provides evidence that task requirements heavily influence the size and prevalence of object-based effects. In Experiment 1 we investigated differences between two commonly used paradigms to investigate object-based attention: A detection task as introduced by Egly et al. [2] and a discrimination task as introduced by Moore et al. [3]. For both tasks, space-based effects were large and prevalent in the majority of subjects, whereas object-based effects were small. However, when simulating sample sizes typically collected in experiments like that and randomly resampling groups of 16 subjects out of the group of 60 we found that object-based effects were more prevalent in the detection than the discrimination task, which indicates that experimenters should use a detection task if they want to maximize the probability of getting significant object-based effects. Of course it needs to be pointed out that the object-based effects are small and hence, close to the noise in RT data. This is especially true for the detection task in Experiment. Therefore, RTs for object-based effects might create a normal distribution of effect sizes across subjects with some significantly above zero and others not, or, as in the case of the discrimination task, even below zero. However, this does not account for any differences between rectangle orientations as found in Experiments 1 and 2. Specifically, analyses of RTs revealed object-based attention effects only for horizontal rectangles for the detection task in Experiment 1 and the discrimination tasks in Experiments 1 and 2. In fact, we failed to find evidence of object-based attention with vertical rectangles in all three experiments.

The effect of orientation is especially surprising as studies on the effects of exogenous (central) cueing on attentional shifting found rather the opposite: Inhibitory effects for invalidly cued targets appearing at locations across the horizontal meridian (e.g., [34]), which would lead to the assumption that object-based effects for horizontal rectangles should be smaller than for vertical rectangles. However, in a more recent study, Botta et al., [35] showed that exogenous (peripheral) and endogenous cueing differentially affect the attentional crossing of the meridian and that meridian crossing only had an effect when endogenous cues were used. This leads to the assumption that exogenous and endogenous cueing are mediated by two separate attentional sub-systems.

The effect of orientation on object-based attention has not (to our knowledge) been described before. Our findings differ from previous studies [2], [4] that found no difference in object-based attention effects between vertical and horizontal rectangles. Many other studies used horizontal and vertical stimuli but did not explicitly test for differences between orientations (e.g., [3], [7], [8], [15]–[17], [21], [36]–[38]). Interestingly, if we had not taken into account differences in orientation in the ANOVAs, and instead pooled the data across orientations, we also would have found small, but significant, object-based attention effects. When resampling random groups of 16 out of the 60 subjects and pooling across orientation, only 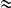
30% of all groups show object-based attention effects in the discrimination task, whereas almost all (92%) groups exhibit object-based attention effects in the detection task. However, in both tasks, the prevalence of object-based effects is larger for horizontal than vertical rectangles when taking into account differences in orientation.

Why does object-based attention primarily occur for horizontal rectangles? One possibility is that attention may be allocated more easily along the horizontal meridian. For horizontal rectangles, attention on the Invalid-same trials was allocated along the horizontal meridian of the visual field, whereas on Invalid-different trials attention was allocated along the vertical meridian. Therefore, if attention was allocated more easily along the horizontal meridian, then RTs measured with horizontal rectangles would be faster on Invalid-same trials than on Invalid-different trials. The layout of the vertical rectangles predicts the opposite pattern of results: RTs would be faster on Invalid-different trials than Invalid-same trials. The hypothesis that information processing is facilitated along the horizontal meridian is consistent with the results of studies of visual search [39]–[44] and change blindness [45]. One recent study [40], for example, investigated the effects of transient covert attention in orientation discrimination, detection and localization tasks, and found that performance in a visual search task along the horizontal meridian was less affected by spatial frequency, set size, or eccentricity than performance along the vertical meridian. Another experiment [41] studied the characteristics of sustained focal attention using a peripheral letter recognition task and found better performance along the horizontal than the vertical meridian.

In the current study, advantages in directing attention along the horizontal meridian might be able to explain general performance benefits for Invalid-same trials on horizontal rectangles and for Invalid-different trials on vertical rectangles. However, they do not explain why the object-based effect for vertical rectangles arises in the detection task but is actually reversed in the discrimination task. This performance difference might be based on the differences between the tasks per se: It has been suggested that discrimination tasks compared to detection tasks are more difficult and demand a more effortful processing of the target [46]. In the present study, such increased difficulty and more effortful processing is revealed by longer RTs and larger error rate for the discrimination task. In detection tasks, the most important process might be to dissociate the target from the cue, whereas in discrimination tasks the cue might simply facilitate target discrimination by helping to select the spatial position where the analysis of the features is going to occur [47]. Therefore, in the current study attention might be kept longer at the cued location in the discrimination task than the detection task so that the features of the symbol that appears after the cue can be properly analysed and accepted or rejected as a potential target. The additional allocation of attention at the cued location in the discrimination task might be longer than the critical temporal window that allows object-based attention to occur and other attentional processes might come into effect, such as an attentional advantage along the horizontal meridian as described above [39]–[43].

In conclusion, the current study shows that object-based attention is not as robust as previously assumed. The occurrence of object-based attention seems to rely not only on the time-course of the cue-target relationship or the predictability of the cue, but also on the nature of the task and other attentional processes. In addition, using bootstrapping, the current study shows the low prevalence of object-based attention effects on the level of single subjects. This illustrates the utility of augmenting statistics on group means with analyses applied to individual subjects. While there is value in averaging results together to understand a population as a whole, that average may not accurately represent the behavior of that group of subjects, let alone the population. For us to create theories of cognition and perception that truly predict behavior, we also need to consider variation on the level of the individual subject.

## References

[1] Posner MI, Snyder CR, Davidson BJ (1980). Attention and the detection of signals.. J Exp Psychol.

[2] Egly R, Driver J, Rafal RD (1994). Shifting visual attention between objects and locations: evidence from normal and parietal lesion subjects.. J Exp Psychol Gen.

[3] Moore CM, Yantis S, Vaughan B (1998). Object-based visual selection: Evidence from perceptual completion.. Psychological Science.

[4] Watson SE, Kramer AF (1999). Object-based visual selective attention and perceptual organization.. Percept Psychophys.

[5] Li X, Logan GD (2008). Object-based attention in chinese readers of chinese words: beyond gestalt principles.. Psychon Bull Rev.

[6] Yantis S (1992). Multielement visual tracking: attention and perceptual organization.. Cogn Psychol.

[7] Avrahami J (1999). Objects of attention, objects of perception.. Percept Psychophys.

[8] Cepeda NJ, Kramer AF (1999). Strategic effects on object-based attentional selection.. Acta Psychol (Amst).

[9] Davis G, Holmes A (2005). Reversal of object-based benefits in visual attention.. Visual Cognition.

[10] Lamy D, Egeth H (2002). Object-based selection: the role of attentional shifts.. Percept Psychophys.

[11] List A, Robertson LC (2007). Inhibition of return and object-based attentional selection.. J Exp Psychol Hum Percept Perform.

[12] Macquistan AD (1997). Object-based allocation of visual attention in response to exogenous, but not endogenous, spatial precues.. Psychon Bull Rev.

[13] Saiki J (2000). Occlusion, symmetry, and object-based attention: comment on behrmann, zemel, and mozer (1998).. J Exp Psychol Hum Percept Perform.

[14] Luo C, Lupianez J, Funes MJ, Fu X (2009). Modulation of spatial stroop by object-based attention but not by space-based attention.. Q J Exp Psychol (Colchester).

[15] Pratt J, Sekuler AB (2001). The effects of occlusion and past experience on the allocation of objectbased attention.. Psychon Bull Rev.

[16] Hecht LN, Vecera SP (2007). Attentional selection of complex objects: joint effects of surface uniformity and part structure.. Psychon Bull Rev.

[17] Abrams RA, Law MB (2000). Object-based visual attention with endogenous orienting.. Percept Psychophys.

[18] Brown JM, Denney HI (2007). Shifting attention into and out of objects: evaluating the processes underlying the object advantage.. Percept Psychophys.

[19] Marrara MT, Moore CM (2003). Object-based selection in the two-rectangles method is not an artifact of the three-sided directional cue.. Percept Psychophys.

[20] Vecera SP, Farah MJ (1994). Does visual attention select objects or locations?. J Exp Psychol Gen.

[21] Goldsmith M, Yeari M (2003). Modulation of object-based attention by spatial focus under endogenous and exogenous orienting.. J Exp Psychol Hum Percept Perform.

[22] Shomstein S, Yantis S (2004). Con_gural and contextual prioritization in object-based attention.. Psychon Bull Rev.

[23] Drummond L, Shomstein S (2010). Object-based attention: shifting or uncertainty?. Atten Percept Psychophys.

[24] Lamy D (2000). Object-based selection under focused attention: a failure to replicate.. Percept Psychophys.

[25] Lavie N, Driver J (1996). On the spatial extent of attention in object-based visual selection.. Percept Psychophys.

[26] Efron B, Tibshirani RJ (1993). An introduction to the bootstrap.

[27] Efron B (1979). Bootstrap methods: another look at the jackknife.. Annals of Statistics.

[28] Mooney CM, Duval RD (1993). Bootstrapping: A nonparametric approach to statistical inference.

[29] Brainard DH (1997). The psychophysics toolbox.. Spat Vis.

[30] Pelli DG (1997). The videotoolbox software for visual psychophysics: transforming numbers into movies.. Spat Vis.

[31] Kirk RE (1968). Experimental design: Procedures for the behavioral Experimental design: Procedures for the behavioral sciences.

[32] Baylis GC, Driver J (1993). Visual attention and objects: evidence for hierarchical coding of location.. J Exp Psychol Hum Percept Perform.

[33] Gottlob LR, Cheal ML, Lyon DR (1999). Timecourse of location cueing effects with a probability manipulation.. Journal of General Psychology.

[34] Rizzolatti G, Riggio L, Dascola I, Umiltá C (1987). Reorienting attention across the horizontal and vertical meridians: evidence in favor of a premotor theory of attention.. Neuropsychologia.

[35] Botta F, Santangelo V, Raffone A, Lupiáñez J, Belardinelli MO (2010). Exogenous and endogenous spatial attention effects on visuospatial working memory.. Q J Exp Psychol (Colchester).

[36] Abrams RA, Law MB (2002). Random visual noise impairs object-based attention.. Exp Brain Res.

[37] Brown JM, Breitmeyer BG, Leighty KA, Denney HI (2006). The path of visual attention.. Acta Psychol (Amst).

[38] Kramer AF, Weber TA (1999). Object-based attentional selection and aging.. Psychol Aging.

[39] Carrasco M, Chang I (1995). The interaction of objective and subjective organizations in a localization search task.. Percept Psychophys.

[40] Carrasco M, Talgar CP, Cameron EL (2001). Characterizing visual performance fields: effects of transient covert attention, spatial frequency, eccentricity, task and set size.. Spat Vis.

[41] Mackeben M (1999). Sustained focal attention and peripheral letter recognition.. Spat Vis.

[42] Montaser-Kouhsari L, Carrasco M (2009). Perceptual asymmetries are preserved in short-term memory tasks.. Atten Percept Psychophys.

[43] Rovamo J, Virsu V (1979). An estimation and application of the human cortical magnification factor.. Exp Brain Res.

[44] Zénon A, Hamed SB, Duhamel JR, Olivier E (2009). Attentional guidance relies on a winner-take-all mechanism.. Vision Res.

[45] Tse PU, Sheinberg DL, Logothetis NK (2003). Attentional enhancement opposite a peripheral ash revealed using change blindness.. Psychol Sci.

[46] Klein (2000). Inhibition of return.. Trends Cogn Sci.

[47] Chica AB, Lupianez J, Bartolomeo P (2006). Dissociating inhibition of return from endogenous orienting of spatial attention: Evidence from detection and discrimination tasks.. Cogn Neuropsychol.

